# Multifaceted Enhancement of L-Leucine-Enriched Ovine Bone Graft: Physicochemical Characteristics and Osteogenic Potential for Improved Guided Bone Regeneration

**DOI:** 10.7759/cureus.64416

**Published:** 2024-07-12

**Authors:** Rahul Koppaka, Khushali K Shah, Subhabrata Maiti

**Affiliations:** 1 Department of Prosthodontics, Saveetha Dental College and Hospitals, Saveetha Institute of Medical and Technical Sciences, Saveetha University, Chennai, IND

**Keywords:** scanning electron microscopy analysis, amino acid, x-ray diffraction, ovine bone, bone graft substitutes

## Abstract

Introduction

Small compounds like L-leucine can boost bone regrowth by blocking certain effects, sparking cell reactions through signaling sequences. This research explored how combining L-leucine with hyaluronic acid on the developed novel graft material affects the bone's ability to conduct bone-building processes.

Material and methods

This study was designed as an in-vitro experiment, where a novel bone graft was formulated by integrating L-leucine with hyaluronic acid and incorporated into a hydroxyapatite-based ovine bone graft material. The sintering procedure was modified to include the amino acid L-arginine. Comprehensive examinations were executed using methodologies such as scanning electron microscopy, X-ray diffractometry, Fourier-transform infrared spectroscopy (FTIR), MTT (3-(4,5-dimethylthiazol-2-yl)-2,5-diphenyltetrazolium bromide) assay, and bone formation assay. These analyses were juxtaposed with the characteristics of the commercially accessible unaltered Bio-Oss, focusing on their physicochemical properties. The properties were compared with a commercially available bone graft material.

Results

The sintered hydroxyapatite/L-leucine graft displayed an interconnected pore structure, indicating that higher sintering and consolidation affected hydroxyapatite, as observed through scanning electron microscopy. X-ray diffraction (XRD) analysis confirmed hydroxyapatite in the sintered ovine bone samples, affirming their suitability for various biomedical applications. In the bone formation assay, optical density (OD) values were 61% for the hydroxyapatite/L-arginine graft, 58% for the Bio-Oss group, and 51% for the control group. The MTT assay, which assesses cell viability and metabolic activity, demonstrated biocompatibility and cell growth for all samples at 24 hours.

Conclusion

The research noted beneficial outcomes by incorporating L-leucine into the novel bone graft material with hyaluronic acid for bone grafting, demonstrating enhanced compatibility with existing bone tissue. However, the specific advantages of this combined approach are not fully known. It is essential to conduct more studies to uncover how this synergy works, assess its prolonged impacts, carry out clinical tests, and enhance the effectiveness of this blend for practical applications in bone graft surgeries.

## Introduction

Despite the widespread utilization of bone graft and substitute materials globally, significant constraints persist regarding the materials currently employed. These issues primarily pertain to allografts, which involve transferring grafting materials between genetically unrelated individuals, and autografts, which require moving grafting material from one part of a person's body to another [1]. Autografts, in particular, result in heightened donor site complications, such as injury, morbidity, deformity, and scarring, due to the necessity for subsequent surgeries [2]. While evidence-based research supporting the indications and safety of these materials is limited, there is a growing market trend toward the innovation of new bone grafting materials and substitutes. Considering these concerns, alongside the global aging population and the increasing demand for bone graft materials, there is a pressing need for extensive research into the development of novel materials with optimal properties for diverse bone grafting techniques [3]. Improving the integration between bone grafts and artificial substitutes with existing bone is crucial in medical situations involving bone defects and healing needs. Current materials face significant challenges in ensuring long-term connectivity with existing bone despite prolonged use. Moreover, expensive growth factors such as bone morphogenetic proteins (BMPs), parathyroid hormone (PTH), and platelet-rich plasma (PRP), while potentially beneficial for bone formation, have limitations and side effects that hinder their widespread application. Consequently, there is a need to explore alternative options [3,4].

One promising avenue is the use of bioinorganic ions, particularly amino acids like L-leucine. These ions show the potential to enhance bone integration without the substantial problems associated with growth factors [5]. Investigating bioinorganic ions could provide a safer and more cost-effective means to improve the integration of bone grafts and substitutes. If these alternative methods prove effective, they could make bone repair and regeneration procedures more accessible by reducing costs and side effects. Although new methods involving bioinorganic ions offer potential solutions to issues with bone grafting and replacements, they require rigorous testing in trials to ensure long-term safety and efficacy. While ensuring grafts merge well with the patient's bone remains challenging, ongoing research into alternatives, such as bioinorganic ions, could lead to simpler, safer, and more affordable approaches to bone healing and regeneration [6].

This study aims to enhance the bone-forming properties of novel ovine bone grafts through modifications. It explores different approaches, such as incorporating a connecting agent and an amino acid, to improve bone substitutes' ability to promote bone growth. The study begins with a hypothesis that there is no significant difference between the modified ovine bone graft with L-leucine and the commercially available Bio-Oss in terms of their physical and bone-forming properties. This hypothesis forms the basis for comparing the modified bone graft's effectiveness with the current standard, aiming to provide valuable insights for developing superior bone-forming substitutes.

## Materials and methods

The present in-vitro study was carried out at a research facility in Chennai, India. The study was approved by the Scientific Review Board (SRB) on 26 August 2022. (SRB/SDC/PROSTHO-20/22/TH-126).

Preparation of ovine bone graft and isolating hydroxyapatite (HA)

**Figure 1:**
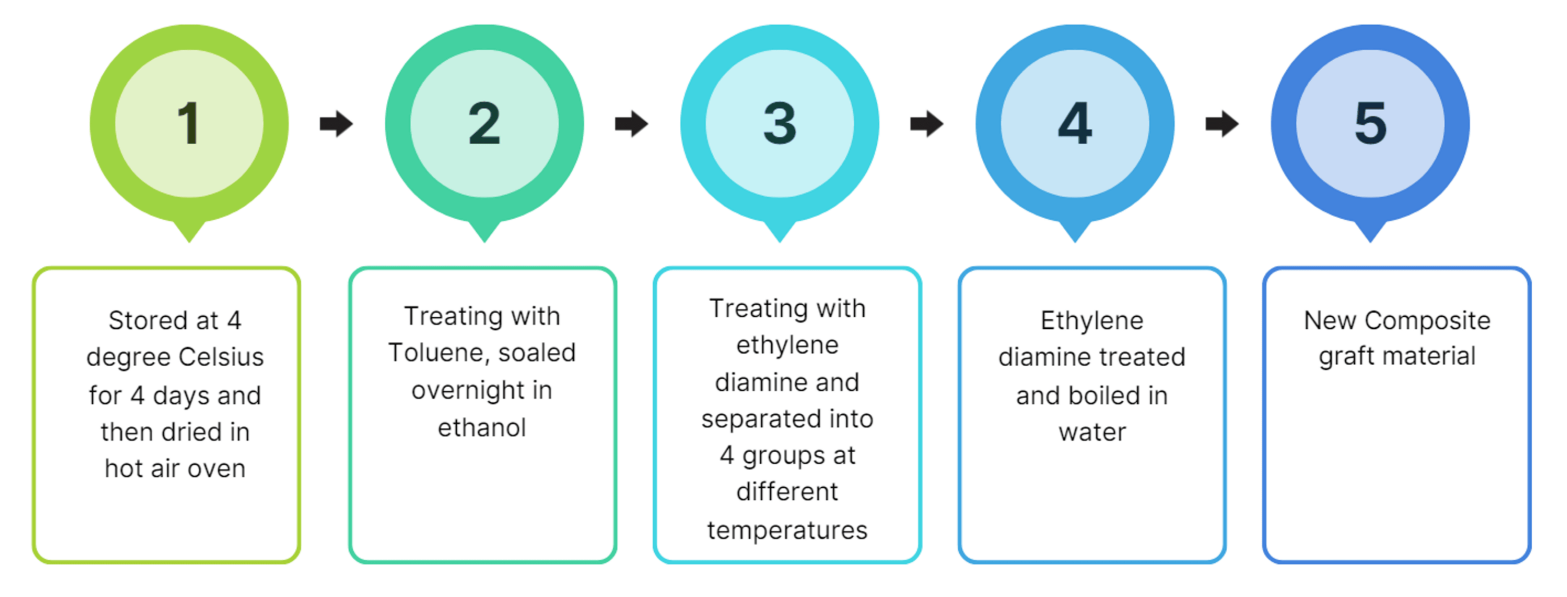
Preparation of hydroxyapatite-based bone graft from ovine bone. Author's creation

**Figure 2:**
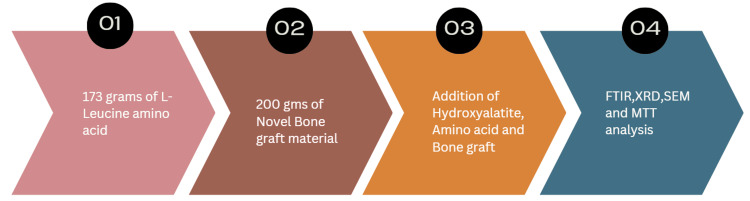
Modification using L-leucine. Author's creation

Samples were obtained from the cortical hind limb femur goat bones collected from the local slaughterhouse located in Chennai, Tamil Nadu. They were cut into smaller pieces as bone comprises both organic and inorganic components. The organic portion contains lipids and proteins, while the inorganic portion includes compounds such as calcium carbonate, HA, calcium oxides, and phosphates. To obtain the inorganic HA, the organic components must first be removed. The preparatory procedure involved extracting the organic constituents: sections of the central region of the ovine femur were cut into large slices, and any remaining soft tissue was carefully excised. To eliminate intrinsic fluids, including marrow and soft tissues, the bone slices were boiled at 100°C for 12 hours. After cleaning, the specimens were stored at 4°C for 12 hours and then subjected to thermal desiccation in a hot air oven at 100°C for another 12 hours. Residual soft tissue and unnecessary material were meticulously removed. Chemical processes were employed to eliminate lipids, adipose content, and proteins using a thermal extraction technique. After a 12-hour immersion phase, the specimens were thoroughly rinsed with ethanol to remove any residual organic solvents. Following the ethanol rinse, the samples were again thermally desiccated in a hot air oven at 100°C for an additional 12 hours. To ensure the complete removal of organic materials, 150 mL of ethylenediamine was used as an additional solvent over a 48-hour period. The specimens underwent two ethanol washes (Figure 1). After the chemical treatment, the samples were sintered at 320°C for three hours. Subsequently, 173 g of L-leucine was added to 20 g of the prepared HA-based bone graft material, with hyaluronic acid used as a binder (Figure 2).

Surface characterization

Samples from both the experimental and control groups underwent comprehensive physicochemical assessments. The analytical methods encompassed scanning electron microscopy (SEM) to thoroughly examine the physical attributes, along with XRD to explore the mineralogical compositions and crystalline integrity. Furthermore, Fourier-transform infrared spectroscopy (FTIR) analysis, MTT (3-(4, 5-dimethylthiazolyl-2)-2, 5-diphenyltetrazolium bromide) assay, and bone formation assay were carried out to assess cellular health and metabolic functions, demonstrating compatibility and proliferation with the living cells.

SEM

Utilizing SEM, various parameters, such as particle size, structural configuration, and surface features, were evaluated. The samples were precisely sized to ensure optimal positioning and stability on the testing platform, concurrently enhancing their conductive properties. Following this preparation, the particulate components underwent a systematic purification process, beginning with a rinse in acetone, followed by deionization using purified water, and concluding with a thorough air-drying step. The final step involved employing a critical point-drying method to eliminate any remaining moisture.

The SEM employed a high-energy electron beam to generate detailed visualizations of the samples. This interaction between electrons and the specimen resulted in the release of secondary electrons, electron backscatter, and specific X-ray signals. Various detectors captured these diverse signals, which were then amalgamated to produce comprehensive visual representations. The inherently short wavelength of the electron beam in SEM images typically results in enhanced clarity and an increased depth perspective, creating a nearly three-dimensional portrayal of the sample. The minute details on the sample surfaces were meticulously examined across various magnification levels, specifically at dimensions of 1, 5, 10, 50, and 100 µm. The JSM-IT800 Nano SEM device, obtained from JEOL Benelux in the Netherlands, was used for this analytical process. Subsequent data analysis involved the utilization of ImageJ software to determine the average particle sizes derived from the SEM visuals.

XRD

XRD analysis was employed to ascertain the mineral compositions and the degree of crystallinity in samples from both the experimental and control groups. The finely powdered specimens were meticulously mounted onto the analysis platform. The examination was carried out using a D8 Advance device obtained from Bruker AXS GmbH in Karlsruhe, Germany, covering an angular range from 10° to 80°.

X-ray emissions primarily originated from a cathode ray tube, which underwent filtration to generate focused monochromatic rays directed at the samples. This process resulted in the formation of diffracted beams, which were subsequently captured, assessed, and quantified. An exact quantity of the sample was thoughtfully placed into an empty holder for the analytical phase. The X-ray wavelength was precisely adjusted to reflect the atomic bond distances within the sample's crystalline structure, creating a distinct pattern indicative of the atomic layout. This analysis involved comparing the observed patterns with established standards to confirm phase uniformity and crystalline quality. The resulting data were visually presented using the Origin software.

Cell Viability - MTT Assay

The specimens were pulverized to achieve a granular consistency suitable for the MTT evaluation, which measures cellular vitality and multiplication rates. In a six-well container, each well was filled with 1 mL of a comprehensive culture solution. Subsequently, a concentration of 0.5 mg/mL of MTT was introduced to the base chamber.

Following these preparations, the plate underwent an incubation phase at a temperature of 37°C for four hours. After this incubation period, the culture fluid was carefully extracted from the designated well. The resulting formazan crystals were then dissolved by adding precisely 100 µL of a DMSO solution into each well. After this addition, the cellular constituents were gently agitated for two minutes to ensure the homogeneous dispersion of the resultant blue-hued reaction derivative throughout the solvent medium. Finally, 100 µL of the tinted DMSO solution from both the inserts and individual wells was dispensed into a fresh 96-well container to quantify cellular vitality. The optical density at a wavelength of 450 nm was determined using a microplate spectrophotometer.

FTIR

The sample was subjected to infrared radiation, and the absorbance spectrum was obtained through Fourier Transform. Important parameters include spectral resolution and scanning range. This method involves measuring the interferogram, from which the spectrum is derived, providing insights into the molecular composition.

Bone Formation Assay - Alizarin Red Staining

In a differentiation medium containing DMEM/F-12 (Dulbecco's Modified Eagle Medium/Nutrient Mixture F-12), 10 mM β-glycerophosphate, 0.05 mM ascorbic acid, and magnesium nanoparticles, the MG-63 cells, osteoclast cells, were grown for 14 days. Calcium deposition was identified using alizarin red staining. The cells were once more stained with 2% alizarin red solution for 10 minutes after two weeks. The cells were subsequently given two washes in 1× phosphate-buffered saline (PBS). Each well received 200 uL of dimethyl sulfoxide (DMSO) for quantitative analysis, which was incubated for one hour. Using a spectrophotometer, the amount of alizarin was determined at 405 nm.

Statistical analysis

All experiments were conducted in at least triplicate, with results presented as mean ± SD. Statistical analysis was performed using SPSS software (Version 26.0; SPSS, Inc., Chicago, IL, USA). Errors for each data point were derived from a minimum of three independent replicate tests, from which the mean and standard deviations were calculated. Comparative measurements between independent data sets were conducted using unpaired t-test analysis, with statistical significance considered for values of p < 0.05.

## Results

HA-based bone graft samples, enhanced with L-leucine, were evaluated in comparison with the commercial bone graft product Bio-Oss. The analysis was conducted using SEM, XRD, FTIR, MTT assay, and bone formation assay.

XRD

**Figure 3:**
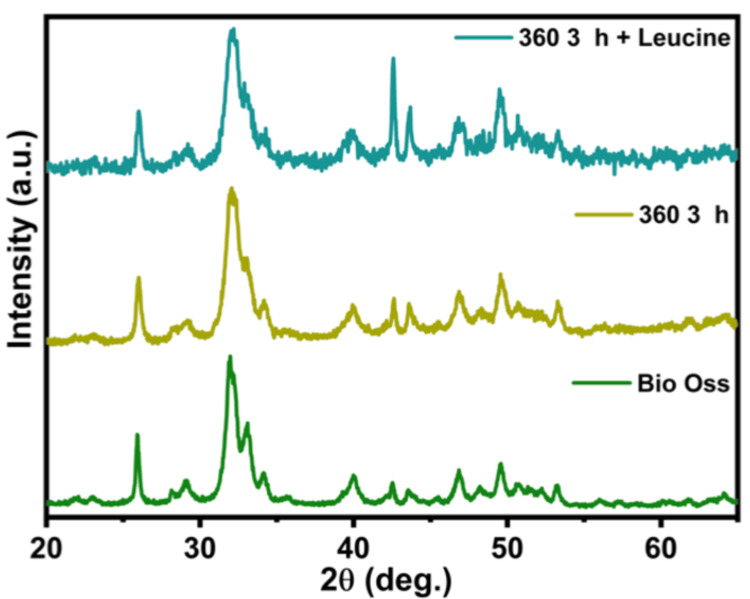
X-ray diffractometry analysis showing peaks of elevated intensity and confirming the presence of hydroxyapatite in commercially available bone graft Bio-Oss, ovine bone graft processed at 360°C, and ovine bone graft + L-leucine.

Upon reviewing the XRD results, distinct crystalline structures of HA and calcium oxide phosphate were identified, along with a minor presence of silica. A distinct high-intensity peak was observed around 31.7° graphically. The XRD pattern of Bio-Oss also displayed three notable peaks, indicating the crystalline form of HA, closely mirroring the observations from the adapted samples. Therefore, the XRD data suggest a high similarity in the crystalline structure between the adapted samples and the composite novel xenograft (Figure 3).

SEM

**Figure 4:**
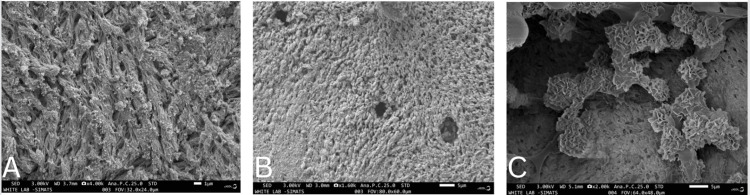
Scanning electron microscopy (SEM) analysis. (A) Bio-Oss. (B) Hydroxyapatite-based bone graft. (C) Hydroxyapatite-based bone graft modified with L-leucine.

The SEM analysis of the composite graft revealed a cohesive structure marked by densely intertwined particles, complemented by noticeable bioactive surface layers on the bone graft's external surface. In contrast, the SEM examination of the commercially available Osseograft bone graft indicated an absence of a consistent pore structure. The fabricated samples exhibited a distinctive pore pattern coupled with a dense composition, showcasing a finely detailed surface configuration compared to the novel bone graft material. The SEM visuals emphasized a remarkably smooth surface consistency with strong particle-to-particle bonding, highlighting the enhanced solidity of the HA matrix (Figure 4).

Cell viability - MTT assay

**Figure 5:**
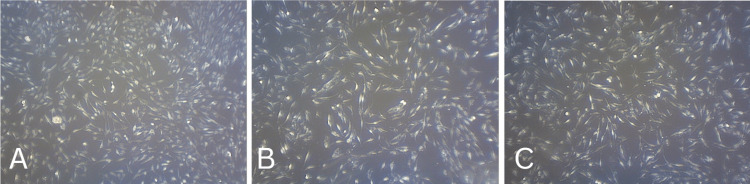
Cell viability at 24 hours showing cell aggregation. (A) Control. (B) 25 µg/mL concentration. (C) 50 µg/mL concentration.

**Figure 6:**
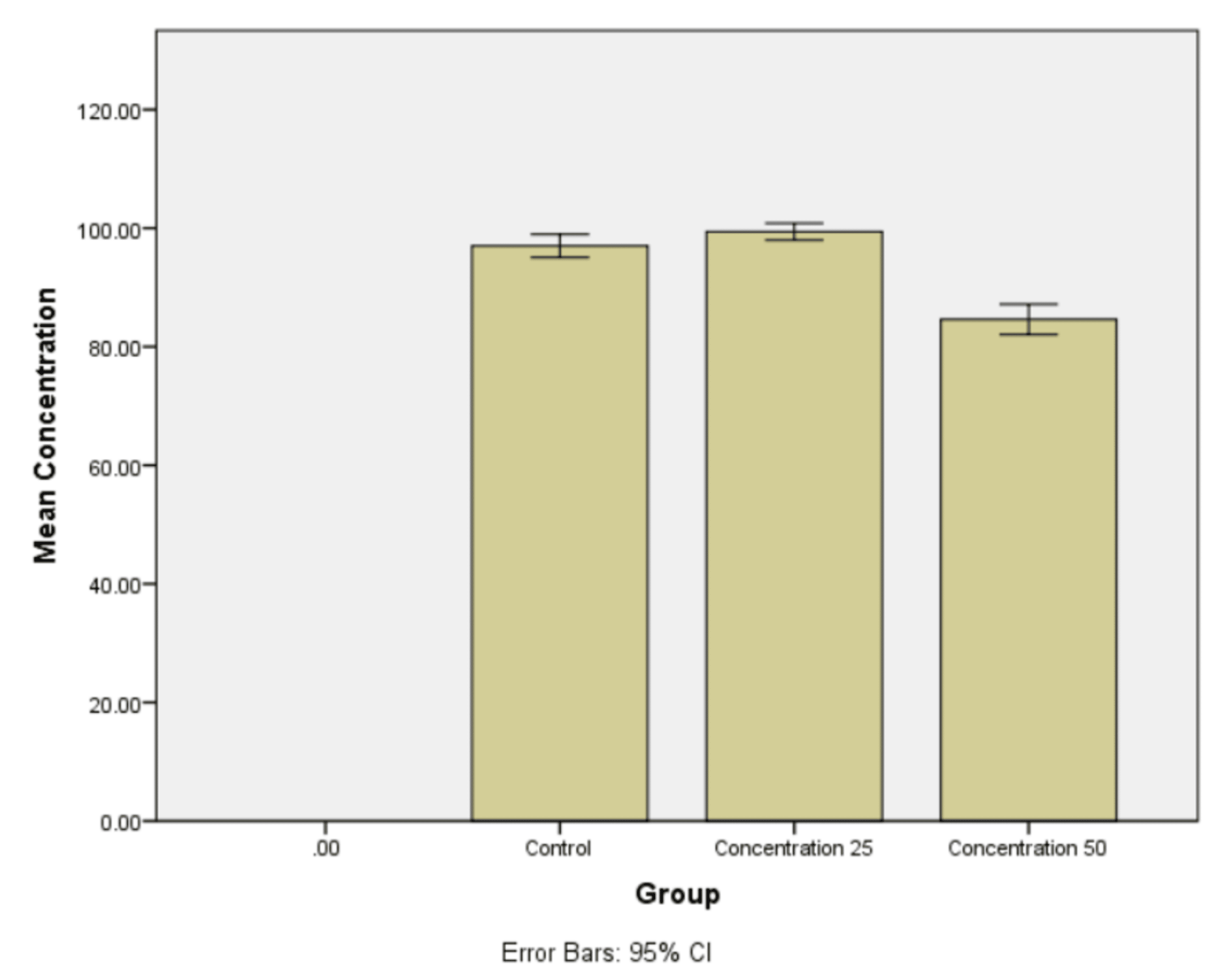
Mean cell aggregation at 25 and 50 µg/mL concentration of bone graft at 24 hours.

The adapted specimens demonstrated a significant increase in MG-63 cell clustering by approximately 1.2 times over 72 hours, contrasting with the two-fold reduction observed with Bio-Oss. Such variations could have a substantial impact and potentially enhance the inherent osteogenic differentiation capabilities of the L-leucine-modified bone graft (Figure 5 and Figure 6).

FTIR

**Figure 7:**
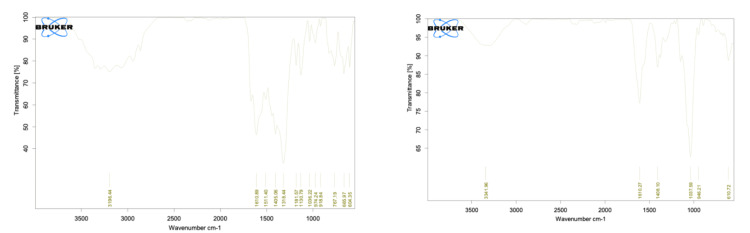
Fourier-transform infrared spectroscopy (FTIR) analysis. (A) Hydroxyapatite-based bone graft modified with L-leucine. (B) Bio-Oss.

The presence of phosphate and carbonate groups characterizes HA. In FTIR analysis, peaks between 1,300 and 1,600 cm^-^¹ confirm these groups, affirming HA's presence. The samples exhibited bands within this range, verifying the presence of HA. Additionally, a peak at 1,037 cm-¹ indicated the presence of L-leucine, suggesting its binding and dispersion within the HA matrix (Figure 7).

Bone formation assay - alizarin red staining

OD values for the bone formation assay were found to be 62% for the ovine bone graft along with L-leucine as the amino acid, whereas it was found to be 58% for the Bio-Oss group and 54% for the control group, thereby showing an increased efficiency of bone formation with that of ovine bone with L-leucine.

## Discussion

Integrating bone grafts or synthetic substitutes into existing bone structures is a tough job in orthopedics and regenerative medicine. Conventionally, bone grafts have been procured from autologous sources, allogeneic donors, or synthetic materials, all pivotal in the remediation of bone defects. Synthetic substitutes have benefits like lower disease risk and can be customized like natural bones [6]. The key to successful recovery is making sure the graft or substitute bonds well with the host bone. If they do not bond properly, it can cause problems like the implant coming loose, making the treatment less effective. Growth factors like BMPs and PTH help bones form but are expensive and can have side effects, so they are not used widely [7]. To tackle these challenges, researchers are looking into bioinorganic ions, like L-leucine, as alternatives to pricey growth factors. These ions seem to help synthetic substitutes integrate with host bones without the downsides of growth factors. By studying bioinorganic ions, researchers aim to create cheaper and safer ways to integrate bones, addressing worries about cost and side effects [7,8]. However, we still need more research to be sure that bioinorganic ions are safe and effective for bone integration. If they prove to be, they could lead to new treatments or make existing ones better, ultimately helping patients and lowering healthcare costs [9].

The exploration of bioinorganic ions marks a big change in orthopedic research, aiming to offer safer and easier-to-access treatments for patients with bone defects [10]. Achieving a seamless union between the graft or substitute and the host bone is crucial for functional recovery and long-term success. Inadequate integration can lead to complications like implant loosening, lack of stability, or even the failure of orthopedic procedures [11]. Despite the demonstrated efficacy of growth factors such as BMP, PTH, and PRP in promoting bone formation and integration, their extensive use is constrained by high costs and potential adverse effects [12]. The combination of L-leucine and hyaluronic acid presents a compelling avenue for enhancing the effectiveness of synthetic graft materials in bone regeneration. L-leucine, a small compound known for its ability to modulate cellular signaling pathways, can play a pivotal role in stimulating bone-forming processes. By incorporating L-leucine into the graft material, we harness its signaling properties to initiate and promote favorable cellular responses crucial for bone formation. This includes activating pathways involved in osteogenesis, such as the mTOR signaling pathway, which regulates cell growth and proliferation [13].

Simultaneously, the inclusion of hyaluronic acid further enhances the graft material's bioactivity. HA is recognized for its biocompatibility, biodegradability, and ability to modulate inflammation and wound healing processes. Its presence within the graft material provides an environment conducive to cell adhesion, migration, and proliferation, facilitating the recruitment of osteogenic cells to the site of regeneration [14]. The observed changes in the structural and chemical properties of the graft material following the incorporation of L-leucine and HA suggest synergistic interactions at play. These interactions likely involve the formation of molecular complexes between L-leucine, HA, and the scaffold matrix, influencing the material's overall architecture and surface characteristics [15]. Such alterations can have profound effects on cellular behavior, including adhesion, proliferation, and differentiation, ultimately contributing to enhanced bone regeneration outcomes [16]. Moving forward, further validation of these findings through in vivo studies is crucial to fully understand the clinical relevance and therapeutic potential of the developed graft material. In vivo models can provide insights into the material's biocompatibility, integration with surrounding tissues, and ability to promote bone regeneration in a physiological environment [17]. Additionally, assessing long-term outcomes, including the material's stability and its impact on bone remodeling processes, will be essential for translating these findings into practical applications in orthopedic surgery and tissue engineering [18]. Overall, the synergistic combination of L-leucine and HA holds great promise for advancing the field of bone regeneration and addressing clinical challenges associated with bone defects and fractures [19]. Amino acids like L-leucine and other bioinorganic ions have attracted attention for their capacity to assist in the integration of synthetic substitutes with host bone structures. These ions show promise in facilitating bonding without the drawbacks associated with expensive growth factors. The investigation of bioinorganic ions presents a potential avenue for more cost-effective and safer integration strategies. If substantiated, these ions could offer a feasible alternative to growth factors, overcoming barriers related to cost and adverse effects [20].

While our study provided valuable insights into the influence of L-leucine on HA derived from ovine bone, it is crucial to acknowledge inherent limitations. Primarily, our focus was largely directed toward sintering parameters, overlooking potential factors such as the intrinsic chemical composition and impurities present in the original material. Future investigations should delve deeper into elucidating the relationship between sintering parameters and the fundamental attributes of the material to achieve a more comprehensive understanding [21].

Furthermore, although our chosen analytical techniques, including SEM, XRD, and FTIR analyses, offered insights into the microstructural complexities, crystalline characteristics, and biological relevance of the sintered HA, there exists potential for the incorporation of more advanced characterization methodologies [22]. Advanced manufacturing techniques, such as additive manufacturing or 3D printing, hold promise for designing intricate structures using sintered HA. Moreover, there is potential to integrate bioactive agents, growth factors, or specialized drug delivery systems to augment the material's regenerative capacities [23]. Additionally, thorough investigations, including prolonged degradation studies and mechanical testing under physiological conditions, would yield crucial insights into the material's long-term durability and structural integrity [24].

Ongoing research is pivotal to validate the efficacy, safety, and mechanisms of action of bioinorganic ions in facilitating bone integration. Rigorous studies can establish their role as viable alternatives or complementary approaches to growth factors. Successful validation could pave the way for the development of novel therapies or enhancements in existing techniques for bone grafting and orthopedic surgeries. This holds the potential to improve patient outcomes and reduce healthcare costs [25]. The ongoing exploration of bioinorganic ions and other innovative approaches represents a dynamic shift in orthopedic research, aiming to address the challenges associated with the integration of bone grafts and synthetic substitutes while fostering safer and more accessible treatment options for patients [26].

## Conclusions

The study found that incorporating L-leucine into graft material with hyaluronic acid improved interaction with native bone and enhanced osteoconductive properties. However, the precise mechanisms through which L-leucine and hyaluronic acid can collectively enhance the bone graft's performance were not fully elucidated by the study's findings. Further research is needed to explore how L-leucine interacts with Bio-Oss and hyaluronic acid at the molecular and cellular levels. Understanding these mechanisms could reveal insights into bone regeneration processes. Long-term studies are essential to ensure the safety and efficacy of these materials. Controlled clinical trials should evaluate their performance in real patient scenarios, assessing outcomes, complications, and overall effectiveness compared to existing treatments. Additionally, research should explore variations in concentrations, ratios, or delivery methods to identify the most effective combinations for bone grafting.
